# GeneTopics - interpretation of gene sets via literature-driven topic models

**DOI:** 10.1186/1752-0509-7-S5-S10

**Published:** 2013-12-09

**Authors:** Vicky Wang, Li Xi, Ahmed Enayetallah, Eric Fauman, Daniel Ziemek

**Affiliations:** 1 Computational Sciences CoE, Pfizer Global Research & Development, Cambridge, Massachusetts, USA; 2 Investigative Toxicology, Drug Safety Research & Development, Pfizer, Groton, USA

## Abstract

**Background:**

Annotation of a set of genes is often accomplished through comparison to a library of labelled gene sets such as biological processes or canonical pathways. However, this approach might fail if the employed libraries are not up to date with the latest research, don't capture relevant biological themes or are curated at a different level of granularity than is required to appropriately analyze the input gene set. At the same time, the vast biomedical literature offers an unstructured repository of the latest research findings that can be tapped to provide thematic sub-groupings for any input gene set.

**Methods:**

Our proposed method relies on a gene-specific text corpus and extracts commonalities between documents in an unsupervised manner using a topic model approach. We automatically determine the number of topics summarizing the corpus and calculate a gene relevancy score for each topic allowing us to eliminate non-specific topics. As a result we obtain a set of literature topics in which each topic is associated with a subset of the input genes providing directly interpretable keywords and corresponding documents for literature research.

**Results:**

We validate our method based on labelled gene sets from the KEGG metabolic pathway collection and the genetic association database (GAD) and show that the approach is able to detect topics consistent with the labelled annotation. Furthermore, we discuss the results on three different types of experimentally derived gene sets, (1) differentially expressed genes from a cardiac hypertrophy experiment in mice, (2) altered transcript abundance in human pancreatic beta cells, and (3) genes implicated by GWA studies to be associated with metabolite levels in a healthy population. In all three cases, we are able to replicate findings from the original papers in a quick and semi-automated manner.

**Conclusions:**

Our approach provides a novel way of automatically generating meaningful annotations for gene sets that are directly tied to relevant articles in the literature. Extending a general topic model method, the approach introduced here establishes a workflow for the interpretation of gene sets generated from diverse experimental scenarios that can complement the classical approach of comparison to reference gene sets.

## Background

Large scale genome-wide omics analysis and advanced sequencing technology have fuelled the generation of gene sets that need to be interpreted and understood quickly and comprehensively. These gene sets are generated from experiments designed to answer various biological questions. Given the complexity of biological systems, it is often required that several different analysis methods are applied to fully understand the functional structure of the gene set. Besides the data-mining techniques that are often used to reduce the dimension of a long gene list to a more human-interpretable size, such as clustering, a very common approach is to compare the gene set to annotated reference gene sets. Ackermann and Strimmer, 2009 gave a comprehensive review [1]. Through statistical testing, the significance of the overlap can be assessed. However, this approach requires a comprehensive collection of manually curated reference gene sets and might fail if the employed libraries are not up to date with the latest research, don't capture relevant biological themes or are curated at a different level of granularity than is required to appropriately analyze the input gene set.

At the same time, the vast biomedical literature offers an unstructured repository of the latest research findings that can be tapped to provide thematic sub-groupings for the gene set under consideration.

Several techniques have been developed to perform information retrieval by processing documents written in natural languages. One of the early widely used approaches was Latent Semantic Analysis (LSA) [2]. It analyzes the word-document association data matrix using singular-value decomposition (SVD) to establish relationships among words and documents. The indexing outcome provides a way to place similar words and documents close to each other. The LSA approach was later extended to a model called Probabilistic Latent Semantic Analysis (PLSA) which models each word in a document as a sample from a mixture model [3]. PLSA represented a more direct approach to model the data than LSA, but its lack of a probabilistic model at the document level led to the development of Latent Dirichlet Model (LDA) [4].

Topic models are algorithms for discovering the main themes that pervade a large and otherwise unstructured collection of documents. Topic modelling algorithms can be applied to massive collections of documents and have been used to find patterns in diversified areas such as genetic data, images, and social networks. In this work we focus on the most popular approach, Latent Dirichlet Model (LDA), to derive topics, but note that many extended algorithms could serve as drop-in replacements in our proposed approach. Briefly, LDA is a probabilistic model based on a "bag-of-words" approach, i.e. it treats a document as an unordered collection of words. It then tries to infer probability distributions over the vocabulary of words thereby defining each of *k *topics. At the same time it determines a mixture of these topic distributions best describing the corpus as a whole. As a result, each document in the corpus can be assigned to one or several topics with different degrees of certainty. Table 1 gives an impression of several topics derived from the literature by listing the words most highly associated with each. Topic models are an active area of research. Blei et al 2012 [5] give a recent overview.

**Table 1:** Topics found for KEGG Metabolic pathways

KEGG Id	Pathway Name	LDA model #	Best Topic	pvalue	Topic words (stemmed)
hsa00010	Glycolysis / Gluconeogenesis	25	Topic11	6.60E-23	strain,acet,mutant,acid,growth,glucos,cerevisia,plant,yeast,enzym,ferment,synthetas,coli,acetylcoa,gene,product,encod,activ,saccharomyc,ac
hsa00020	Citrate cycle (TCA cycle)	10	Topic7	1.87E-08	mitochondri,activ,enzym,oxid,aconitas,b5,dehydrogenas,reductas,cytochrom,malat,inhibit,mitochondria,reduct,nadhcytochrom,b5r,alphaketoglutar,cytosol,kgdhc,citrat,inactiv
hsa00030	Pentose phosphate pathway	25	Topic7	1.82E-12	enzym,activ,pfk,fructos,inhibit,ph,purifi,atp,subunit,concentr,pfk1,kinet,degre,aldolas,alloster,affin,kda,molecular,appar,purif
hsa00040	Pentose and glucuronate interconversions	25	Topic19	1.20E-20	glucuronid,human,ugt,liver,microsom,ugt2b7,activ,udpglucuronosyltransferas,ugt1a9,ugt1a6,ugt1a4,substrat,ugt1a1,isoform,ugt1a3,ugt1a10,valu,acid,express,format
hsa00051	Fructose and mannose metabolism	25	Topic19	1.58E-10	fructos,fructokinas,activ,enzym,pmm,sugar,plant,phosphoryl,khk,sucros,substrat,mannos,l,km,character,glucos,ketohexokinas,gene,clone,metabol
hsa00052	Galactose metabolism	25	Topic14	3.05E-09	mutat,diseas,patient,defici,gene,caus,allel,clinic,storag,case,acid,glycogen,type,muscl,identifi,disord,or,genet,polymorph,lysosom
hsa00053	Ascorbate and aldarate metabolism	50	Topic2	1.60E-13	glucuronid,human,ugt,liver,microsom,activ,ugt2b7,udpglucuronosyltransferas,ugt1a9,substrat,ugt1a1,valu,ugt1a6,ugt1a3,ugt1a4,metabol,enzym,kinet,microm,inhibit
hsa00062	Fatty acid elongation	10	Topic9	1.53E-08	peroxisom,enzym,dehydrogenas,hydratas,activ,acid,thiolas,betaoxid,enoylcoa,acylcoa,fatti,coli,3ketoacylcoa,prolin,p5cdh,substrat,liver,coa,oxid,catalyz
hsa00071	Fatty acid metabolism	50	Topic19	1.76E-16	enzym,activ,substrat,structur,dehydrogenas,residu,bind,site,catalyt,acid,mutant,dhdps,coli,specif,reaction,inhibit,form,studi,kinet,differ
hsa00100	Steroid biosynthesis	10	Topic4	6.45E-10	cholesterol,acat2,ester,acat,lipas,acat1,esteras,intestin,lipoprotein,lipid,cholesteryl,ldl,acyltransferas,liver,mice,cel,pancreat,plasma,bile,acid

The table shows the GeneTopics results for KEGG metabolic pathway. Only the best topic with the smallest pvalue is shown. Many of the topic words are either part of the labelled pathway name or are closely related terms.

In this work, we propose to leverage topic models in a specific way to support the identification of biologically coherent subgroups of genes in an input gene set. Our proposed method has distinct advantages to current approaches. Namely, we don't rely on possibly outdated or irrelevant curated libraries, but can access the latest research to detect sub-groupings. Moreover we can directly provide literature reference for the inferred topics and their associated genes, thereby greatly facilitating the inevitable follow-up work by computational biologists to explain the association of single genes with a certain biological process or context.

Topic models have been studied extensively in the literature and improved inference methods based on relaxed model assumptions are continuously being proposed [5]. Also, LDA-based methods have been applied to the analysis of gene expression experiments [6]. While the underlying mathematical formalism is the same, these latter approaches do not relate findings to a corpus of text at all and are, thus, unrelated to our method.

To our knowledge, topic models have not been applied systematically to the interpretation and annotation of experimentally derived gene sets. Lu et al, 2006 [7] analyzed the semantic coherence of LDA-derived topics on a corpus for 300 proteins linked to the literature via the UniProt database [8]. In contrast to our work, the authors do not establish a method to derive a mapping of subgroups of genes to topics and don't provide a gene-topic score, but rather stop at the conclusion that the resulting topics reflect relevant literature topics and establish semantic coherence based on pre-specified Gene Ontology sets [9]. More recently, Wang et al [10] have proposed the integration of controlled and normalized terms, such as gene symbols or compound IDs, into the LDA inference process to improve performance by avoiding ambiguity. They do not explicitly focus on the interpretation of an input gene set in terms of the topics as we do in our method. However, our method might benefit from normalizing known terms in PubMed abstracts.

The remainder of the paper will detail our proposed method, discuss validation of our implementation and finally outline results on three biological datasets from transcriptomics and genetics experiments.

## Methods

**Figure 1:**
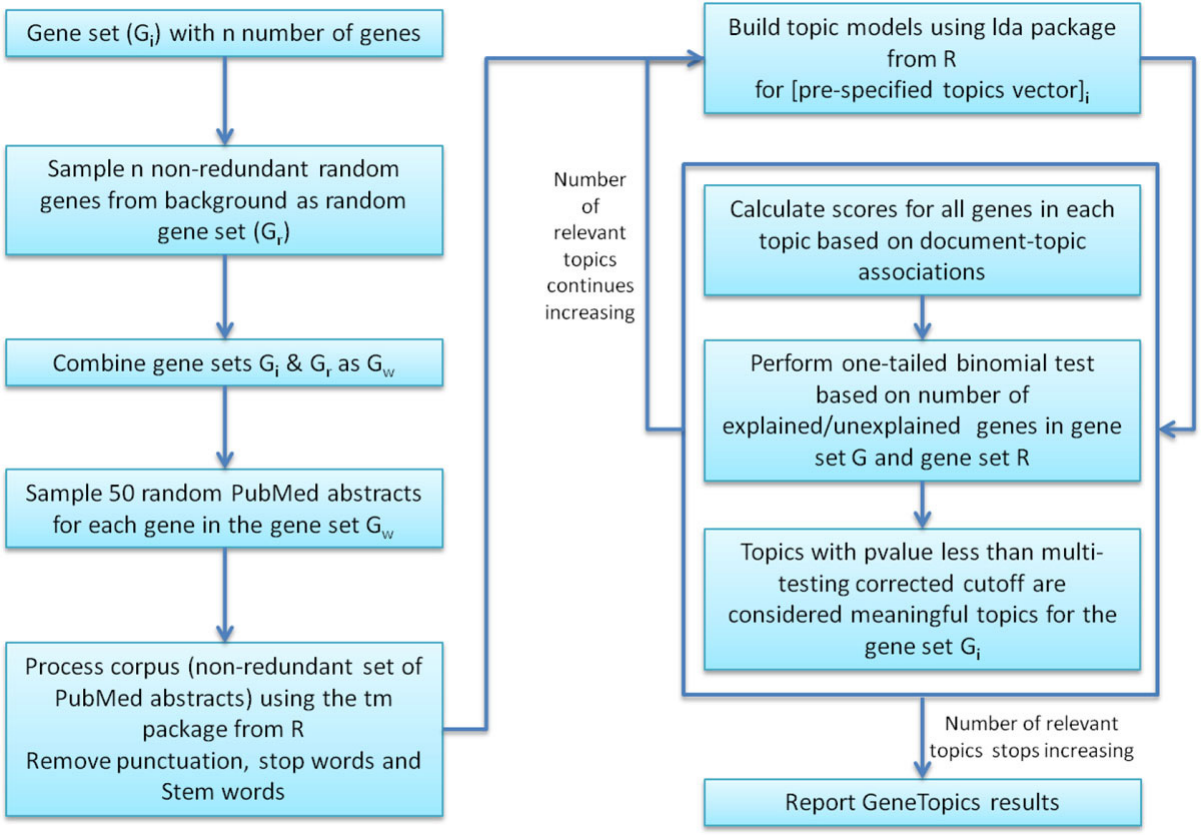
**GeneTopics algorithm workflow**. This diagram provides an overview of the steps involved in taking a list of genes and generating the list of relevant topics with related genes and PubMed references.

Using topic models, our approach takes an input set of genes, G_i_, and generates relevant topics, T, associated with subsets of genes based on a constructed corpus. Figure 1 depicts a flow diagram of our method which consists of several main steps, namely corpus compilation, topic model inference, gene score enrichment, estimation of the appropriate number of topics to infer and reporting of results.

### Compilation of a corpus with embedded noise for significance testing

Given an input list of genes, G_i_, we first *randomly *select an equal number of genes, G_r_, from the same background gene population and add them to the input gene set to define our working gene set, G_w_={g_1_, ..., g_N_}. The random set is needed later in the pipeline to triage topics not specific to G_i_. As a base corpus of text, we chose all abstracts in the PubMed database of biomedical literature which contains approximately 15 million citations as of 2013. Although it is desirable to use all the available abstracts for our analysis, we noticed that many of the earlier publications have limited texts in the abstracts and out-of-date information. To address this issue, we decided to limit the publication to be ones that are published after 1995 and have high confidence scores with the associated genes. This gives us over 1.5 million citations to work with. To associate genes in G_w _with documents in the corpus, we rely on a method that detects all abstracts mentioning a given gene and scores the occurrence based on syntactic and linguistic features such as place and frequency of occurrence resulting in a relevancy score for each gene-document pair [Phoebe Roberts, personal communication on LitMS gene-document index tool]. Other potential sources of such associations are the MeSH and GeneRIF databases [http://www.ncbi.nlm.nih.gov/gene/about-generif]. Based on this association, we pick a representative sample of relevant documents for each gene in G_w _to form our corpus consisting of documents D={d_1_, ..., d_M_} for topic modelling. As the number of publications associated with a gene could vary and we aim for equal representation of each gene, we define the number of documents as a parameter in our method with the default value set to 50. This means that the title and abstract of up to 50 papers from each gene will be used to assemble a corpus. This default number is empirically chosen based on both the computation time and to avoid over-representation of documents for certain well-studied genes. Using the '*tm*' package in R [11], we perform standard text-mining operations to remove stop words and punctuations followed by the application of the Porter stemming to reduce words in the abstracts to their stems. We then tokenize the corpus to form a term-document matrix for both original and stemmed words. The stemmed version is for the subsequent topic model analysis while the version with original words is used to reverse the stemmed words so the end result is more interpretable. Depending on the gene set size, the average size of vocabularies is around 15K.

### Topic model inference

Topic model inference is a commonly used approach for uncovering the main themes from large yet unstructured collection of documents. In our method, we used the topic model implementation in the R '*lda' *package [http://cran.r-project.org/web/packages/lda/] implementing the classic LDA approach suggested by Blei et al [4]. The probability distribution inferred by LDA specifies the probability of occurrence for each word in the corpus in documents of each topic. Based on this distribution, we can assign each word in each document to its most likely topic and define the following matrix:

$$\begin{array}{c} {\left[\text{Word Count Matrix}\right]}_{\text{i},\text{j}}=\text{ number of times a word in Document }{\text{d}}_{\text{i}}\text{is}\\ \;\;\;\;\;\;\;\text{assigned to topic }{\text{T}}_{\text{j}}\text{ by the model}. \end{array}$$

Normalizing this matrix per column, gives us the Topic Proportion Matrix as

$$\begin{array}{c} {\left[\text{Topic Proportion Matrix}\right]}_{\text{i},\text{j}}=\text{ proportion of words in Document }{\text{d}}_{\text{i}}\\ \;\;\;\;\;\;\;\;\;\;\;\;\text{assigned to topic }{\text{T}}_{\text{j}}\;\text{by the model}. \end{array}$$

**Figure 2:**
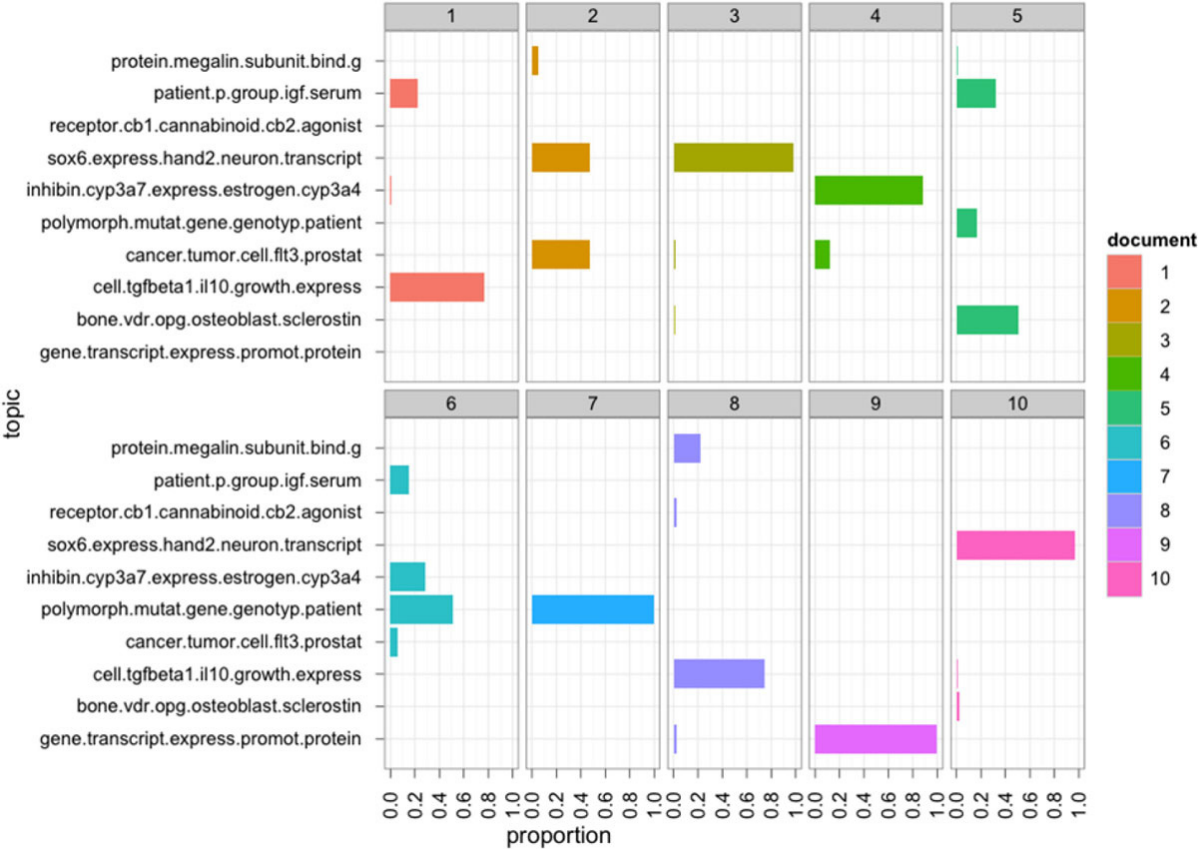
**Topic proportions of documents**. This plot shows the topic proportions for the first 10 documents of a corpus from a LDA model run. The number 10 was specified as the number of topics to build the model and the top 5 terms is used to represent the topics found. Each document is associated with 1 to many topics and the proportion is calculated based on number of words assigned to the topic divided by total number of words for the document.

For example, if document d_1 _has 100 words and 40 of them were assigned by the topic model to topic t_1 _and 60 of them were assigned to topic t_2_, then for document d_1_, the proportion score is 0.4 for topic t_1 _and 0.6 for topic t_2 _and 0 for the rest of the topics. This matrix reflects the assumption of LDA that each document is associated with a mixture of topics (Figure 2). We will use this Topic Proportion Matrix to assign genes to topics in the following.

### Gene assignment and enrichment calculation

Since our corpus was derived from a list of genes and the association between genes and the documents is available, each gene is associated with a number of documents and therefore rows in the topic proportion matrix. We consider for each gene g_i _all Documents D_gi _with indices {x_1_, ..., x_n_} that are associated with the gene and define a Gene Topic Matrix as

$${\left[\text{Gene Topic Matrix}\right]}_{\text{i},\text{j}}=\text{ ma}{{\text{x}}_{\text{k}}}_{=\text{1},..,\text{n}}\left(\left[\text{Topic Proportion Matrix}\right]{\text{x}}_{\text{k},\text{j}}\right).$$

This matrix captures the association of each gene with each topic. Note that the maximization operator in the above definition emphasizes the multi-functional role many genes play in many different biological contexts. This is also consistent with the nature of publications which often focus on one specific biological aspect of an experimental system (e.g. oxidative phosphorylation or apoptosis) and discuss associated genes. Alternative options (such as summarization or average) tended to underestimate gene topic associations and led to score distributions tightly clustered around the mean.

As we know the composition of the analyzed gene set G_w_, we may now use it to distinguish topics that are relevant to the initial gene set (G_i_) as compared to the randomly sampled background gene set (G_r_).

**Figure 3:**
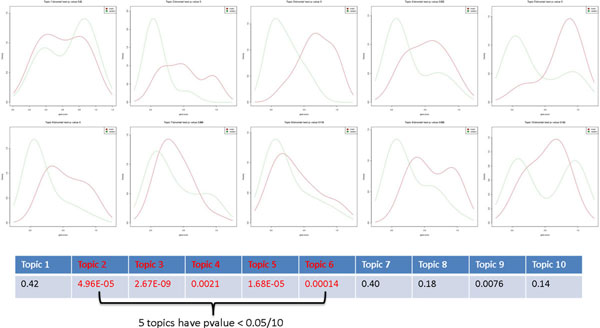
**Perform enrichment test to identify topics relevant to gene set**. Different gene score distribution can be distinguished by the statistical test and only the models where higher gene scores (> 0.5) are significantly larger than random are selected.

To determine the relevance of a topic to a subset of G_i_, we perform a statistical test of the null hypothesis that scores for G_i _and G_r _have the same distribution. Figure 3 gives various examples for score distributions in one of our validation settings. Intuitively, we prefer settings in which the score distribution for genes in G_i _is shifted to the right as compared to G_r_. Following this intuition, we prototyped several tests which assess whether the two sets of scores are likely drawn from the same underlying distribution (e.g. using a Kolmogorov-Smirnov test) or whether the means of the score distribution are the same (e.g. using a Wilcoxon-Rank-Sum test [10]). However, in both cases we found that biologically irrelevant topics were flagged as statistically significant. This situation arose mostly when the scores for G_i _were clearly enriched for higher scores as compared to G_r_, but the mean of the scores was still low, i.e. below 0.5. As a consequence, we decided to use a binomial test for proportions on discretized score distributions.

Note that applying a threshold *t *to the Gene-Topic-Matrix induces (potentially overlapping) sets of genes to be associated with each topic. In the following, we will use a threshold *t *= 0.5, but this parameter can be adjusted to focus on stronger or weaker associations of genes to topics. For a given topic and threshold *t*, we can directly estimate the proportion of genes from the background set with strong associations as

$$\text{prop}\text{\_}\text{random}=\left(\#\text{genes in }{\text{G}}_{\text{r}}\text{with scores}>0.\text{5}\right)/\left(\#\text{genes in }{\text{G}}_{\text{r}}\right).$$

We can then test whether the score distribution for the input gene set G_i _exhibits the same proportion with respect to threshold *t *= 0.5 using a one-tailed binomial test.

In addition, we correct the resulting p-values for multiple testing using the conservative Bonferroni-correction. This is important as the number of topics depends on the size and nature of the gene set and we are interested in controlling the false-positive rate regardless of the number of topics tested. The topics that have a multiple-testing corrected p-value less than 0.05 are considered relevant to our input gene set G_i_. Figure 3 shows that different gene score distribution can be distinguished by the statistical test and only the models where higher gene scores (> 0.5) are significantly larger than random are selected.

### Determining an appropriate number of topics

**Figure 4:**
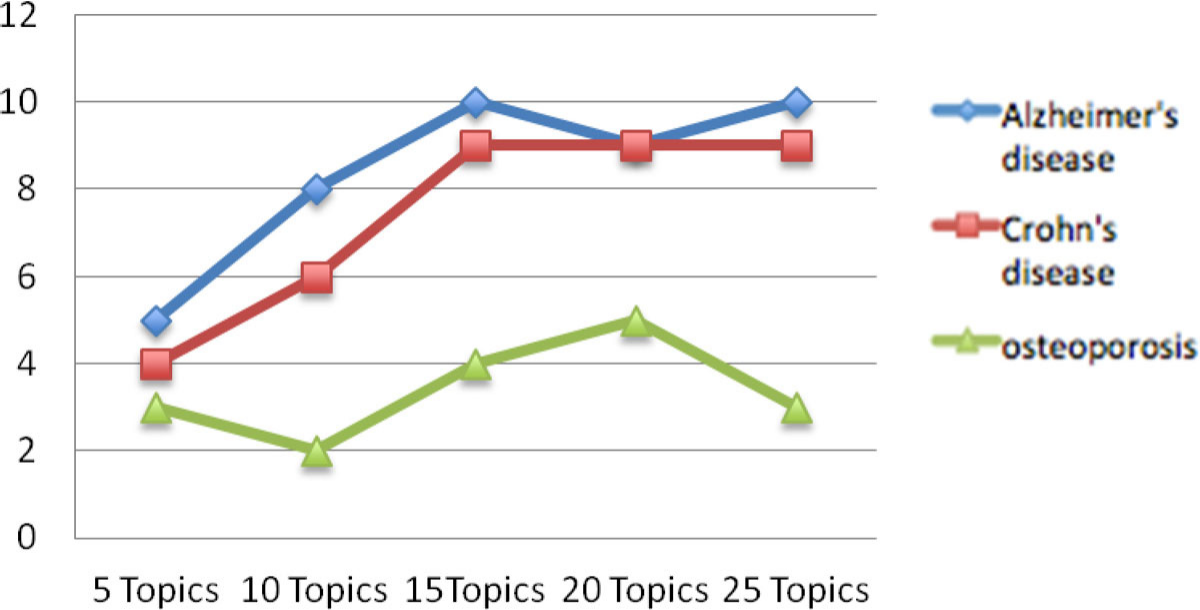
**Empirically determine the appropriate number of topics**. This plot demonstrates the number of relevant topics found in LDA models built with different number of topics. The appropriate number used for building the LDA model is determined when the number of relevant topics found stops to increase or starts to decrease. The data here shows that the 15-topic LDA model yielded 10 relevant topics for Alzheimer's disease gene set and 9 for Crohn's disease gene set. For osteoporosis, 3 relevant topics were found in the 5-topic LDA model.

Like many other similar algorithms, the number of topics to be inferred by LDA needs to be specified as a parameter. We apply a parameter search to determine a reasonable number of topics for our purposes. This iterative process runs LDA with the number of topics pre-specified as [5,10,15,20,25,30,40,50,75,100] and infers topics relevant to the input gene set G_i _as described above. We continue inference until the number of topics deemed significant stops to increase or starts to descend from the maximum. Figure 4 shows the number of relevant topics found for the 3 disease related gene sets - Alzheimer's disease, Crohn's disease and Osteoporosis during the iterative process of estimating the appropriate number of topics by our method. For both Alzheimer's disease and Crohn's disease, the optimal number for fitting an LDA model for the respective corpus is 15 while the number is 5 for the corpus of Osteoporosis.

### Report GeneTopics results

The relevant topics found by our method for a gene set are visually represented by the top 20 related terms (words). By glancing through the words of each of the topics, a scientist can start to develop an intuition about the biological functions the gene set is related to similar to assessing the results of popular gene set enrichment analysis approaches. To help establish a more granular view on the subgroups of the gene set, our method reports genes that have scores > 0.5 for each topic. PubMed IDs associated with each gene in a topic are also listed for in-depth study of underlying articles.

## Results

To evaluate the method's performance in automatically annotating a gene set, we used previously annotated gene sets from well-known and publicly available resources. Specifically, we used gene sets that are annotated metabolic pathways from the KEGG database [13] and genetic associations from the GAD collection [14].

### Validation using metabolic pathways

We downloaded and constructed gene sets in the Metabolism category from the KEGG PATHWAY Database [13]. There are 226 human-specific metabolic pathways and 6101 genes are involved in these pathways. The size of the gene sets ranges from 12 to 1138 with a median of 59 genes. We ran the described workflow for all 226 gene sets with parameters set to 50 articles to select for each gene in G_i _and pre-defined topic numbers to [10,25,50].

**Figure 5:**
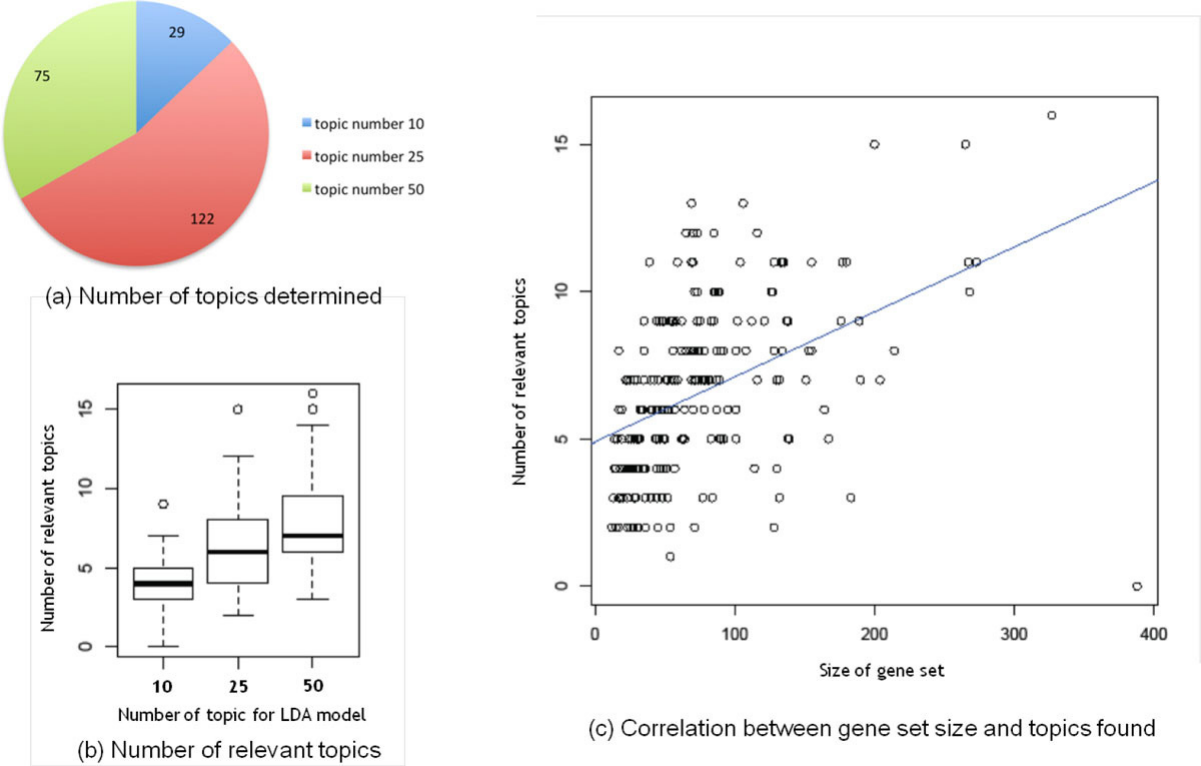
**KEGG Metabolic pathway gene sets**. The GeneTopics algorithm was applied to 236 KEGG metabolic pathway gene sets. (a) Three pre-defined number of topics were used in the validation for each gene set, the pie chart shows the distribution of gene sets for which the optimal number was determined. (b) Number of relevant topics increases as the number is used to build the optimal LDA model. (c) The size of the gene set and the number relevant topics found have a positive correlation. All these results are consistent with expectation intuition and indicate that the GeneTopics algorithm operates properly and is suitable for large-scale analyses.

Out of the 226 genesets, our method determined for 29 gene sets the number of topics to be 10, for 122 genesets 25 and for 75 genesets 50 topics were determined (Figure 5a). The number of topics that pass the statistical threshold of 0.05 showed steady increase as the number of topics was fitted to build the model (Figure 5b). As this is the first analysis of using topic model to find biological themes for gene sets, we compared the number of topics found against the number of genes in the gene set. As expected there is a positive correlation between the two sets of numbers (Figure 5c).

We now examine the relevant topics found by our approach for each of the gene sets. Table 1 shows the topics with the best p-value along with the top 20 scoring terms for 10 gene sets (full table please see additional file 1). Clearly, our method recovers - in a completely unsupervised way - many of the words occurring in the label for the pathway in virtually all cases. This establishes that for well-studied gene sets like metabolic pathways the method is able to recover key information on the gene sets.

### Validation using genes genetically associated with disease

To test if the method is robust enough to find topics for genes that are being grouped as a set due to a different biological aspects, we used data collected in the genetic association database (GAD) [14]. Here, genes are annotated based on their association to genetics diseases in GWAS studies. We constructed 157 genetic disease relevant gene sets that are associated with 1455 genes. Several different parameters were used to systematically test the gene sets: (a) run the workflow with the default background gene set of 13767 in our gene-document index collection (b) run the workflow with the GAD genes as the background gene set and (c) randomly mix 3 GAD gene sets and attempt to recover relevant topics.

Table 2 shows the partial results of best topics found for GAD gene sets using all genes as background (full table please see additional file 2). Many terms associated with GWAS studies such as polymorphism, association, genotype, frequency...etc appear as the top words in the topic with the best p-value. Although such topics are expected for gene sets with genetic association, it is the functional aspects of the gene sets related to the diseases that are more interesting. To overcome this problem, we randomly selected genes from the 1455 GAD genes when simulating the background null distribution in the workflow. Table 3 shows the results of best topics found for the modified workflow. Many of the best topics are much more consistent with the expected disease terms. We also noticed that in many of the cases, the topics found were related to different molecular aspects of the studies for the disease. For example, some of the topics clearly point to the transcriptional regulation of the disease while other topics are focused on finding related to the enzymatic reactions or signal transductions.

**Table 2:** Topics found for GAD genetic diseases gene set

GAD gene set	LDA model #	Relevant number of topics	Best Topic
esophageal cancer	20	7	genotyp,polymorph,risk,associ,cancer,95,gstm1,gene,or,ci,control,studi,allel,gstt1,p,patient,genet,gstp1,frequenc,signific
kidney cancer	15	2	polymorph,xrcc1,risk,repair,cancer,genotyp,dna,95,associ,ci,gene,allel,variant,control,patient,suscept,breast,frequenc,case,ratio
Alzheimer's disease	15	10	polymorph,genotyp,associ,allel,risk,gene,patient,p,popul,genet,95,variant,diseas,haplotyp,diabet,subject,frequenc,ci,snps,control
blood pressure, arterial	20	11	polymorph,genotyp,gene,associ,allel,p,risk,variant,hypertens,patient,genet,subject,popul,95,haplotyp,frequenc,ci,variat,studi,pressur
carotid atherosclerosis	10	6	eno,endotheli,ace,oxid,rat,inhibitor,nitric,group,renal,p,inhibit,day,treatment,synthas,after,vascular,arteri,heart,angiotensin,enzym
hypertension	20	14	arteri,rat,angiotensin,pressur,vascular,hypertens,heart,cardiac,receptor,renal,blood,ang,increas,ace,endotheli,ventricular,express,after,at1,muscl
cirrhosis	10	5	iron,transferrin,receptor,tfr,hfe,ferritin,cell,stfr,method,uptak,serum,assay,status,sampl,antibodi,marrow,recycl,blood,concentr,defici
hepatitis c, chronic	10	7	polymorph,il12b,genotyp,associ,allel,risk,gene,frequenc,patient,haplotyp,genet,diseas,suscept,p,popul,asthma,ci,il12,variant,95
longevity	15	7	polymorph,genotyp,gstm1,gene,risk,associ,gstt1,cancer,allel,frequenc,genet,95,null,ci,patient,control,variant,popul,individu,p
diabetes, type 2	20	14	polymorph,associ,genotyp,gene,allel,p,risk,diabet,variant,patient,subject,genet,popul,snps,type,frequenc,haplotyp,95,2,studi

This table shows the best topic found by GeneTopics for gene sets derived from GAD (Genetic Association Database). Many of the best topics found are related to the common origins of the GAD gene sets.

**Table 3:** GeneTopics results for GAD gene sets with GAD genes as background

GAD gene set	LDA model #	Relevant number of topics	Best Topic
esophageal cancer	20	7	polymorphism, genotype, risk, associated, 95, cancer, or, gene, ci, allelic, studies, gstm1, patients, controls, p, gstt1, frequency, genetic, population, variants
kidney cancer	15	2	cyclin, d1, expressed, cancer, tumor, cells, carcinomas, breast, cases, cycle, correlated, survival, invasive, cdk4, patients, gastric, proteins, oncogene, overexpression, associated
Alzheimer's disease	15	10	diseases, alzheimers, ad, amyloid, abeta, app, tau, brain, ide, platelets, gammasecretase, parkinsons, ps1, pd, protein, fe65, precursor, lrrk2, titin, beta
blood pressure, arterial	20	11	renal, hypertension, ace, kidney, pressure, aldosterone, angiotensin, uroguanylin, ace2, blood, cyp11b2, sodium, enzyme, intestinal, excretion, urinary, guanylin, ae1, kae1, peptides
hepatitis c, chronic	10	7	chemokin,cell,t,ccr5,express,rant,mcp1,cytokin,macrophag,il10,il8,cxcr1,monocyt,receptor,infect,immun,hiv1,virus,secret,neutrophil
longevity	15	7	patients, il6, diseases, ad, clinical, therapy, serum, level, p, values, group, outcome, survival, healthy, cytokines, predicted, prognostic, stages, correlate, transplantation
diabetes, type 2	20	14	insulin, glucose, diabetes, leptin, adipose, muscle, obese, expression, metabolism, adiponectin, fat, fatty, mice, islets, increase, mrna, tissues, adipocytes, skeletal, levels

This table shows the best topics found by GeneTopics using the GAD genes as the background. Comparing to results in table 2, the best topics are much more consistent with the expected disease terms.

**Figure 6:**
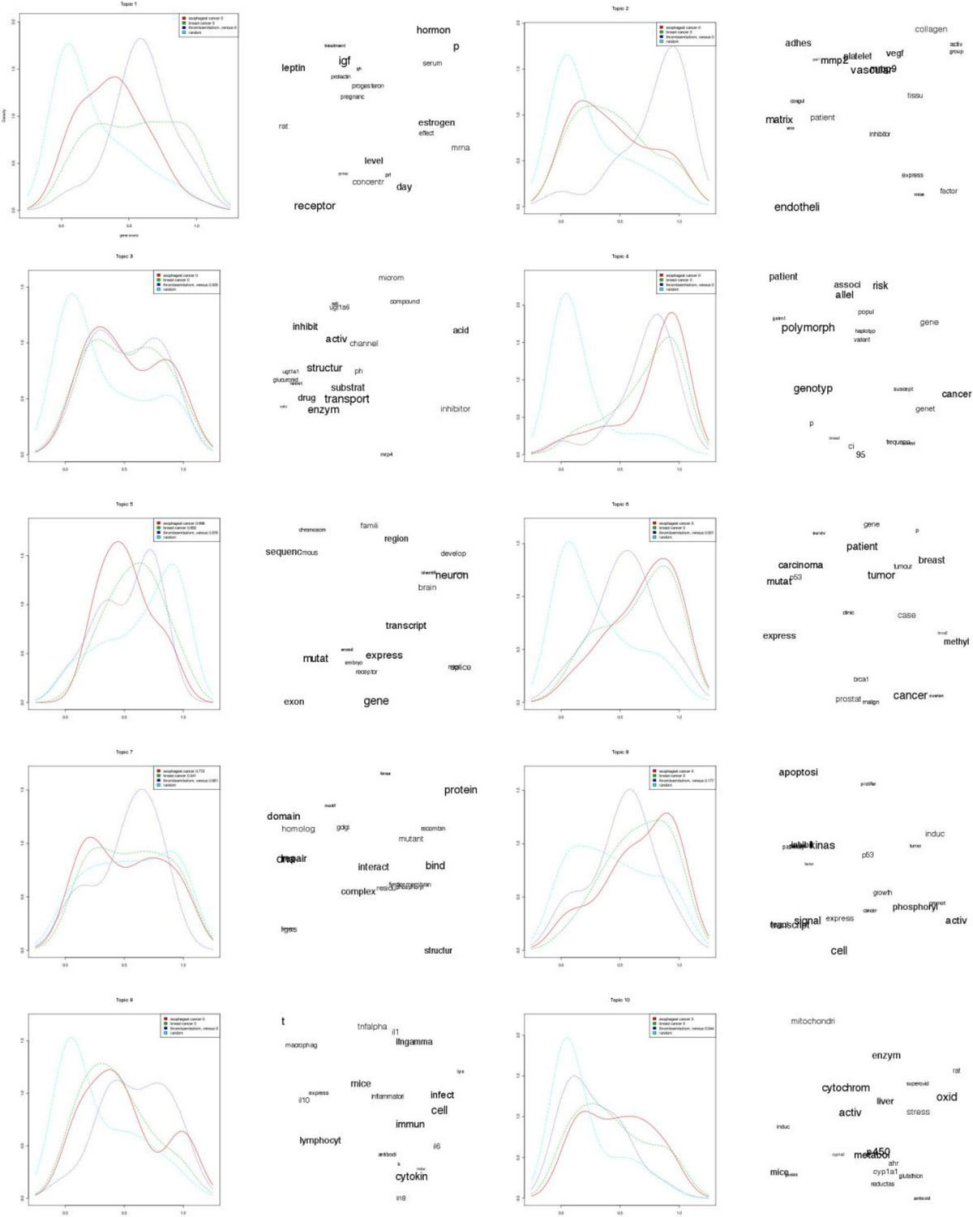
**Results of mixing multiple gene sets**. GeneTopics is able to uncover topics that are associated to subgroup of the gene sets. The density plots on the left show gene score from the 3 embedded gene sets - [esophageal cancer - red line], [breast cancer - green line] and [thromboembolism, venous - purple line] and 1 random gene set [cyan line]. The topic words on the right are shown in tag cloud format.

To test if our approach can decipher functional subgroups embedded in the gene set, we constructed 10 gene sets by randomly selecting 3 GAD gene sets and merging them into one gene set for topic model analysis. Figure 6 shows the gene score distribution and topic words for a gene set made up of 3 GAD gene sets - [esophageal cancer], [breast cancer] and [thromboembolism, venous] and 1 random gene set. As we used all genes as the background pool, the topic 4 shows the distinction between all 3 gene sets from the random gene set. Gene set [thromboembolism, venous] scored best in the topic 2 as the genes in this gene set have high scores associated with the topic words. The two gene sets related to cancer - esophageal cancer and breast cancer are best described in the topic 6 as the keywords such as breast, brca1 and carcinoma appear in the top 20 topic words.

### Testing the workflow with random gene sets

As a negative control for our method, we applied the method on randomly constructed gene sets. As the randomly constructed gene sets should contain no unified themes, we expect that no real topics can be found for these gene sets. To generate such gene sets, we permuted the association between GAD disease term and genes but kept the original GAD gene set size. We then submit all the random gene sets for GeneTopics analysis. To reduce the computation time, we set the LDA model number to be 10 for all gene sets instead of iteratively trying to determine the best number. Our method did not select any significant topics, indicating that it is well able to detect and deprioritize randomly constructed gene sets.

### Case studies I - Human islet gene expression data

Gene expression data in human Islets from 54 non-diabetic and 9 diabetic donors [15] were analyzed and the top 324 genes differentially expressed in T2D donors (using nominal p < 0.05 and absolute value of ratio > 1.3 as cutoff) were used as the input for our algorithm. The method extended the analysis of differentially expressed genes to predicted functions (Table 4). 11 Topics were found as significant for the gene set. Topic 9 contains many terms relevant to diabetes and is associated to many known T2D-associated genes such as key genes in glucose metabolism disorder (ABCC8, CEL, GLP1R, IL6, INS, PCK1, RBP4, SCD, SLC30A8). Topic 19 is related to vasculature, a process intimately interwined with diabetes[16]. The key factor VEGF is among the representative terms for the topic. Since there are several immunological terms in the topic, a second round of analysis may yield more granularity to the genes associated with this topic. Many studies have linked diabetes to neurodegenerative disease such as Alzheimer. Topic 1 identified the topic and many known genes such as 5ht (SLC6A4) and TPH2 in the topic words. It will be interesting to explore the pathological roles of genes associated with this topic in both diabetes and Alzheimer's diseases.

**Table 4:** Topics found by GeneTopics for Human islet gene expression data

Topic	Topic words	pvalue
Topic 19	endothelial, cell, vascular, expressions, activation, cytokine, receptor, il6, inhibitor, vegf, il1, growth, factor, inflammatory, matrix, angiogenesis, metalloproteinases, macrophage, inhibit, lung	9.32E-12
Topic 5	polymorphisms, associated, genes, allele, genotypes, variants, risk, p, genetic, diabetes, snps, patient, haplotype, population, subjected, frequency, variation, susceptibility, 95, snp	4.18E-08
Topic 8	rat, effects, increases, receptor, renal, after, groups, h, antagonists, day, 1, p, heart, mice, cardiac, mrna, kidney, injection, treatment, activation	4.96E-08
Topic 18	patient, p, groups, serum, levels, plasma, clinical, concentration, measured, correlation, disease, treatment, aged, n, sample, healthy, years, women, blood, vs	2.24E-07
Topic 9	insulin, glucose, pancreatic, diabetes, islet, liver, secretion, mice, acid, metabolism, obesity, fatty, betacells, ghrelin, rat, hepatitis, increases, lpa, lipid, fat	7.89E-06
Topic 6	cancer, tumor, expressions, cell, carcinoma, breast, genes, gastric, prostate, invasion, tissues, line, mrna, patient, lung, tumour, colorectal, malignant, normal, human	0.000149
Topic 20	expressions, growth, mrna, cell, collagen, activin, cartilage, matrix, factor, cultured, tissues, day, skin, igf, keratinocytes, tgfbeta, human, chondrocyte, differentially, fibroblasts	0.000153
Topic 14	binding, structure, domain, residues, enzymes, activation, site, complex, peptide, acid, substrate, protein, affinity, sequences, crystallization, form, amino, interaction, purified, nterminal	0.000228
Topic 1	added, oxidase, pedf, alzheimers, tau, apod, 5ht, nox4, polyamines, tph2, disease, ros, apolipoprotein, 5ht1b, brain, apo, d, nadph, serotonin, app	0.003461
Topic 3	developed, expressions, cell, mice, axon, embryonic, genes, differentially, embryos, signaling, neural, mutants, regulated, transcription, mouse, migrating, neurons, pattern, prox1, factor	0.00916
Topic 16	cell, t, immune, infection, receptor, expressions, mice, nk, antibody, antigen, virus, b, lymphocytes, cd8, cytokine, response, tcells, cd4, macrophage, human	0.042753

### Case study II - gene expression data from mouse cardiac tissue

In this example we looked at gene expression profiling of mouse cardiac tissue originally published by [17]. The experiment compared response to Isoprenaline induced hypertrophy (pathologic injury) to response to swim induced hypertrophy (physiologic adaptation). The gene expression changes resulted in 8 and 6 topics for the isoprenaline and the swim groups, respectively (Table 5a and 5b). First, we looked for any terms that would indicate the involvement of the organ *heart *in each group. In the isoprenaline group Topic 4 expressed terms such as heart, cardiac, injury, induced, treatment all which were particularly interesting as they closely relate to the experimental perturbation of isoprenaline induced cardiac injury. In the swim group, however, Topic 12 shows heart related terms such as muscle, endothelial, cardiac, collagene, heart, vascular and hypertrophied, all of which are interestingly consistent with the experimental perturbation of physiologic cardiac hypertrophy. Other topics in the isoprenaline group point to themes around kinase signaling (topic 17), oxidative stress (topic 7), tissue remodeling (topic 12), protein metabolism (topic 5) and muscle glucose and lipid metabolism (topic9). These topics are consistent with mechanisms would expect in response to pathologic challenge to the heart muscle. Other topics for the swim group on the other hand reflected a more benign profile such as glucose and energy metabolism (topic 7), cell cycle/growth/proliferation (topic 13), gene, transcription and protein regulation (topics 3 and 6) and cytoskeleton and cellular organelles (topic 4). Overall, the method uncovers - in an unsupervised way - relevant biological topics and provides literature references for follow-up.

**Table 5:** Topics found by GeneTopics for mouse cardiac tissue gene expression data.

Topic	topic words	Common Theme(s)
Topic 8	cancer, tumor, expressed, breast, carcinomas, prostate, cells, invasion, gastric, tissues, patients, tumour, metastasis, lung, p, malignant, normal, colorectal, lines, correlating	Carcinogenesis
Topic 4	rat, after, increase, p, effect, heart, groups, days, cardiac, h, receptor, mice, mrna, injury, injection, levels, 1, induced, treatment, lung	Treatment induced cardiac injury
Topic 9	insulin, muscle, glucose, diabetes, skeletal, metabolism, mice, adipocytes, obese, adipose, acid, increase, fatty, expressed, lipid, islets, fat, mitochondrial, glycogen, rat	Muscle glucose/lipid metabolism
Topic 17	kinases, activation, phosphorylation, signaling, receptor, pathway, tyrosine, inhibition, cells, regulated, induced, transcript, nfkappab, protein, factor, inhibitor, promotes, bind, growth, stimulated	Kinase/phosphorylation signaling
Topic 14	polymorphisms, associated, genotyping, allele, risk, genes, variants, p, genetic, patients, populations, snps, disease, 95, haplotypes, susceptibility, frequency, ci, schizophrenia, subjects	Genetic effects
Topic 7	oxidation, activation, liver, enzyme, glutathione, metabolism, cytochromes, copper, oxidase, antioxidant, microsomal, rat, ho1, p450, stress, oxygenation, heme, species, cysteine, ros	Oxidative stress
Topic 12	matrix, cells, collagen, bone, expressed, tgfbeta, cartilage, extracellular, growth, tissues, metalloproteinases, vascular, chondrocytes, fibroblasts, tgfbeta1, cultures, endothelial, mmp2, fibronectin, osteoblasts	Tissue remodelling
Topic 5	structural, bind, residue, domain, enzyme, peptide, activation, substrates, site, complex, form, interacts, purified, protein, affinity, crystal, acid, kda, amino, catalytic	Amino acid, peptide and protein metabolism
**Topic**	**topic words**	**Common Theme(s)**
Topic 7	liver, increased, mice, rat, oxide, levels, insulin, metabolism, p, day, glucose, mrna, plasma, after, acid, mitochondrial, effects, transported, hepatitis, diet	Glucose/energy metabolism
Topic 13	cells, apoptosis, activity, kinase, phosphorylation, inhibition, cyclin, expression, growth, induced, signal, regulation, inhibitors, pathway, proliferation, p53, proteins, death, increased, genes	Cell cycle/growth and proliferation
Topic 3	transcripts, binding, genes, factors, proteins, splicing, promoter, rna, interaction, regulation, nuclear, element, repress, domain, translation, activity, complex, mrna, site, histones	Gene and protein regulation processes
Topic 12	muscle, expression, endothelial, cardiac, collagene, cells, heart, vascular, mice, matrix, skeletal, tissues, bone, smooth, vessels, vegf, rat, hypertrophied, decorin, increased	Muscular (skeletal or cardiac) and blood vessel changes
Topic 6	genes, sequence, expression, encoding, cloning, cdna, chromosome, human, region, proteins, transcripts, genomic, mouse, amino, homolog, acid, strains, conserved, exons, plants	Very generic gene, sequence, expression...
Topic 4	proteins, domain, membrane, interaction, binding, complex, actin, cells, golgi, signal, regulation, ubiquitin, transported, localization, mitochondrial, vesicle, function, gtpase, required, trafficking	Cytoskeleton and cellular organelles

The topic words are used to associate gene subsets to common themes.

(a) Isoprenaline Topics

(b) Swim Topics

### Case study III - GWAS data on Metabolite levels

In this case study, we consider results from a genome-wide association study (GWAS) that identified 90 genetic loci associated with blood metabolite concentrations in a normal population [18]. We extended the loci to include 411 nearest genes and used our method to analyze this gene set, potentially supporting the researcher to identify the true causal gene in the locus. Table 6 shows the 8 topics found by our method. Topic 16 quickly points to the fact that many of the potentially causal genes are linked to rare severe disorders of metabolism - an interesting fact that was also discussed in Suhre et al, 2012. Topic 17 shows that our method in an unsupervised way analyzes an input data set from many different angles. In this case, it points out that a number of the papers talking about the implicated genes are related to metabolites measured in the clinic. In a pharmaceutical setting, this will provide quick pointers to articles on current biomarker practice for those metabolites. The majority of the genes related to topic 28 are enzymes, clearly a key biological aspect of genes related to metabolite concentrations, and the topic 38 included many genes that have come up in previous GWA studies. In addition, terms found for topic 4 indicate that another unifying theme for the input gene set is metabolite transport. Many of the transporter genes were found to be associated with the topic such as the genes from solute carrier family SLC22A1 and SLC6A10. Finally, topic 5 is associated with genes related to blood metabolites which again points directly at literature relevant to the subject under study. As a conclusion, the uncovered topics facilitate the grouping of the 411 input genes into relevant categories. It gives high-level overviews through the topic words for each topic and enables the researcher to quickly dive into the relevant literature for more in-depth follow-up.

**Table 6:** Topics found by GeneTopics for GWA studies measuring metabolite level changes

Topic	Topic words	pvalue
Topic 23	liver, human, glucuronidation, activities, metabolic, microsomal, enzyme, p450, ugts, ugt1a9, ugt1a1, udpglucuronosyltransferase, ugt1a6, substrate, ugt1a7, cyp3a5, ahr, drug, hepatitis, metabolites	2.29E-19
Topic 28	enzyme, activities, substrate, structure, acid, cytochrome, residues, purified, reaction, inhibited, oxidation, formed, inhibitor, dehydrogenase, electron, ph, crystal, coli, binding, production	1.73E-08
Topic 22	apo, lipoprotein, apolipoproteins, cholesterol, lipids, plasma, hdl, triglyceride, lipase, apoa, apoc, ldl, tg, level, metabolic, el, cetp, particles, density, hepatitis	3.55E-05
Topic 17	patients, or, treatment, study, clinic, groups, diseases, therapy, tested, years, method, assessed, evaluate, response, cases, who, p, rate, after, treated	7.63E-05
Topic 5	level, increased, p, rat, after, activities, effect, groups, concentration, control, decreased, or, mrna, days, compared, not, h, plasma, blood, higher	0.000175
Topic 4	transporter, uptake, renal, acid, organization, cation, kidney, cotransporter, membranes, amino, taurine, choline, intestinal, rat, apical, anions, octn2, tubule, microm, drug	0.000581
Topic 38	associated, polymorphism, allele, genotype, gene, variants, risk, study, patients, genetic, p, or, population, control, diseases, 95, snps, haplotype, frequency, significance	0.004274
Topic 16	mutations, patients, gene, deficiency, syndrome, caused, family, diseases, disorders, phenotype, identified, genetic, analysis, clinic, reporter, defective, severely, cases, exons, affected	0.011144

## Discussion

Overall our novel method provides a quick and useful way to analyze any input gene with respect to coherent topics in the literature. As a result, each coherent topic is described by its top constituent words to give a quick overview of the contents and is annotated with the genes most strongly associated with it.

One of the most compelling advantages of this approach is its independence from curated gene set libraries. Clearly, the biomedical literature is growing at an enormous pace and it is likely that curation efforts are outpaced. Furthermore, as our examples show topics can point to surprising aspects of the gene set under consideration. While pre-defined gene sets on metabolic pathways are probably reasonably well curated and updated (e.g. in the KEGG database), it is unlikely that gene sets are available capturing all genes controlling metabolites measured in the clinic - a topic discovered for our Metabolomics GWAS.

The approach is quite general and is not custom-tailored to a specific data modality such as transcriptomics data. Here we investigated its use for genetics data as well as transcriptomics data, but we expect it to work equally well in other settings, if coherent topics for the genes exist in the literature.

Our method utilizes two key algorithms that are treated as black boxes, i.e. an association of genes to documents in the corpus and an implementation of a topic model algorithm. Note that the exact nature of either approach is not crucial and we expect improvements in either compartment method to improve the results of our workflow. In fact, especially topic model algorithms have been the subject of intense research over the past years. In our experience, the limitation to a "bag of words" approach is a disadvantage in the implementation we used. Probably an algorithm exploiting phrase structure of documents would lead to topic descriptions that are even easier to interpret by the biologist. Other limitations of the methods largely results from the data (documents) that are analyzed. In our application, if a gene is not mentioned with certain features in the corpus, then the topic will not include such findings. Although we tried to remove non-specific topics by using the random gene set and appropriate background set, we sometimes see very general topics found that are not specific or with enough granularity to the studied genes. Also, the LDA approach does not consider relationships among topics.

To avoid bias when prioritizing topics for the studied genes, the background gene population should be restricted to a set sharing the common themes as the studied genes. For instance, when studying a gene list consisting of kinases with different functions, using all human genes as the background will recover a common topic of "kinase function". However, using all kinases as the background to draw from will focus the method on orthogonal aspects of the kinases under consideration. We found this "parameter" of the method of great importance as it can draw out contrasts of relevance to the customer and reduce the amount of statistically significant, but uninteresting topics.

Finally, we found that the development of an interactive user interface would probably be beneficial for the acceptance of topic model based methods in a larger community. As it stands, results are communicated in hyperlinked spreadsheets. While this works reasonably well, a more interactive approach with potential recursive invocation of the algorithm on subsets of genes should be beneficial to let the user guide the search for topics more quickly into a biologically relevant direction.

## Conclusions

In this work, we presented a novel method that combines ideas from gene set enrichment with topic model inference. Our algorithm is able to quickly and comprehensively identify topics in the literature that a biologist should consider when interpreting a gene list resulting from a given experiment. Especially, in the case of larger result sets that are hard to assess manually, the grouping into literature topics can be a great asset.

We found that the topics are usually reasonably well described by the currently employed topic algorithm, but we see potential for improvement here. As topic model inference is an area of active research, we expect improvements in inference (e.g. of relevant phrases instead of words) to directly improve the usability of our method.

In future work, we plan to develop an interactive UI for the algorithm to enable the user to guide iterative or recursive application of the method to the most interesting topics and leverage ongoing research in the area of topic models to arrive at topics that soften the bag of words assumption and describe topics with sentence phrases for better interpretability.

Overall, we found that the current algorithm was able to recover topics coherent with pre-defined gene sets concerned with metabolic pathways from KEGG as well as genetically associated disease genes from GAD. In our tests with gene sets resulting from experiments, the results were able to quickly point to relevant literature and group the large set of genes into manageable subsets. Especially, the notion of unexpected topics (e.g. clinical metabolites) seems relevant and can complement the classical approach of comparison to reference gene sets.

## Competing interests

The authors declare that they have no competing interests.

## Authors' contributions

VW and DZ designed and implemented the method and analyzed the validation data. LX, EF, AE analyzed results for biological datasets.

## Supplementary Material

Additional file 1 The file contains the full table of topics for 226 KEGG gene sets. Table 1 is a subset of this file. Click here for file

Additional file 2 The file contains the full table of topics for 157 GAD gene sets. Table 2 is a subset of this file. Click here for file

## Declarations

The publication costs for this article were funded by the Computational Sciences CoE at Pfizer, Inc.

This article has been published as part of *BMC Systems Biology *Volume 7 Supplement 5, 2013: Selected articles from the International Conference on Intelligent Biology and Medicine (ICIBM 2013): Systems Biology. The full contents of the supplement are available online at http://www.biomedcentral.com/bmcsystbiol/supplements/7/S5.
